# Genetic predisposition to milder forms of COVID-19 may provide some resilience to head and neck cancers

**DOI:** 10.3389/fonc.2024.1384061

**Published:** 2024-07-08

**Authors:** Boxuan Han, Minghong Sun, Yanming Zhao, Ancha Baranova, Hongbao Cao, Shaokun Liu, Xixi Shen, Lizhen Hou, Jugao Fang, Meng Lian

**Affiliations:** ^1^ Department of Otorhinolaryngology Head and Neck Surgery, Beijing Tongren Hospital, Capital Medical University, Beijing, China; ^2^ Department of Otorhinolaryngology Head and Neck Surgery, Qingdao Municipal Hospital, Qingdao, China; ^3^ School of Systems Biology, George Mason University, Manassas, VA, United States; ^4^ Research Centre for Medical Genetics, Moscow, Russia

**Keywords:** COVID-19, head and neck cancer, Mendelian randomization, genetic factors, causal relationship

## Abstract

**Introduction:**

The impact of the COVID-19 pandemic on head and neck cancer (HNC) has been suggested, but the causal relationship remains unclear.

**Methods:**

We explore this connection by utilizing the Mendelian randomization (MR) approach applied to publicly available genome-wide association study (GWAS) summary datasets for COVID-19 and HNC. The datasets included critical COVID-19 (13,769 cases, 1,072,442 controls), hospitalized COVID-19 (32,519 cases, 2,062,805 controls), SARS-CoV-2 infection (122,616 cases, 2,475,240 controls), and HNC (2,131 cases, 287,137 controls). Mechanistic underpinnings of the causal relationships identified by MR analysis were explored through functional annotation augmented by AI-based literature data mining.

**Results:**

Surprisingly, a genetic predisposition to contracting a milder form of COVID-19 substantially reduced the risks of developing HNC (OR: 0.52, 95% CI: 0.35–0.78, p = 1.42E-03), with no significant association between genetic liability to severe COVID-19 and the risk of HNC detected. Additionally, our findings highlighted 14 genes linked to SARS-CoV-2 infection, potentially playing a protective role in the context of HNC. These genes include *OAS1, LOC107985887, BCL11A, DPP9, LOC107984685, LINC02326, MUC4, NXPE3, IFNAR2, LZTFL1, LOC105372437, NAPSA, LOC105376622, LOC107986082*, and *SLC6A20*.

**Conclusion:**

Our study emphasizes the protective role of the genetic liability to milder COVID-19 in reducing the risk of HNC while refuting a causal relationship between severe COVID-19 and HNC.

## 1 Introduction

Head and neck cancer (HNC) comprises a diverse group of tumors originating in structures within the head and neck region, including the oral cavity, pharynx, larynx, and salivary glands. It ranks as the 6th most common cancer worldwide, constituting approximately 4% of all cancer cases (1). HNC demonstrates a higher incidence in men compared to women, with a male-to-female ratio of around 2:1. Global variation in HNC incidence is influenced by adverse exposures such as tobacco and alcohol use, as well as viral infections like human papillomavirus (HPV) (1) and family history.

COVID-19 has created a global pandemic. Notably, the course of COVID-19 illness varies, with milder cases being common and ambulatory, and severe cases leading to respiratory failure and death, especially in older individuals and those with pre-existing conditions (2, 3). While the transmission of the SARS-CoV-2 virus occurs through the air, the receptivity of human tissue to this virus is not limited to the respiratory tract. For example, the oral cavity epithelium as well as that in salivary glands are vulnerable to SARS-CoV-2 infection through the expression of angiotensin-converting enzyme 2 (ACE2) cell receptors (4). In oral pathologies spanning from head and neck squamous cell carcinoma (HNSCC) (5) to periodontitis (6), amounts of ACE2 displayed on the surface of cells change, thus influencing cellular susceptibility to coronavirus infection. Moreover, earlier studies suggested that COVID-19 may negatively influence the prognosis and outcomes of already existing HNC (7–9). In particular, one of the studies undertook an investigation of the causal relationships between the infection with SARS-CoV-2 as well as critical or hospitalized COVID-19 and 33 categories of cancers by Mendelian randomization (MR) to show the genetically predicted oropharyngeal cancer, among many other types of tumors, has no causal association with COVID-19 severity, hospitalization, or susceptibility (10). Nevertheless, the same research group presented the results of the inverse-variance-weighted MR model as suggestive evidence that the genetic predisposition towards critically ill COVID-19 is connected to an elevated susceptibility to several forms of malignancies, including esophageal cancer. Moreover, the same group was successful in establishing an ESCC risk signature based on sub-clusters of SARS-CoV-2-related genes (SCRGs) discerned by single-cell RNA sequencing (11). Therefore, further studies of the causal effects of COVID-19 on HNC remain warranted.

This study’s primary objective was to investigate the potential causal impact of COVID-19 on HNC at the genetic level, employing Mendelian randomization (MR) analysis. We aimed to test the hypothesis that the genetic predisposition to various forms of COVID-19 may be correlated with the risk of developing HNC. MR, as an epidemiological method, is the method of choice when assessing whether the potential association between exposure and outcome is causal. MR utilizes genetic variants, randomly assigned to the embryo at birth, as instrumental variables (IVs) to minimize the influence of confounders or reverse causations in such investigations (12). Given that conducting a Randomized Clinical Trial (RCT) to dissect the influence of COVID-19 on the risk of HNC is impossible, we have evaluated the potential impact and causal associations of COVID-19 on HNC using the MR-guided analysis of the largest COVID-19-related GWA datasets collected to date and the HNC outcomes sourced from FinnGen R9.

## 2 Methods

### 2.1 GWAS summary datasets

In this study, publicly accessible summary results of GWAS for COVID-19 and HNC were utilized. The COVID-19 GWAS datasets, specific to the European population and released on April 8, 2022, were obtained from the COVID-19 Host Genetics Initiative (HGI). These datasets encompassed critical COVID-19 (13,769 cases and 1,072,442 controls), hospitalized COVID-19 (32,519 cases and 2,062,805 controls), and SARS-CoV-2 infection (122,616 cases and 2,475,240 controls) (13). Additionally, the HNC dataset, comprising 2,131 cases and 287,137 controls (phenocode: C3_HEAD_AND_NECK_EXALLC), was sourced from FinnGen R9 (https://r9.risteys.finngen.fi/) (14). All participants in the datasets were of European origin, and prior to analysis, the alleles and effects of these datasets were harmonized.

### 2.2 MR analysis

The primary analysis for Mendelian randomization (MR) employed the inverse-variance weighted (IVW) technique as major analysis method, complemented by the incorporation of the weighted median and MR-Egger approaches from TwoSampleMR (15). Genetic variants associated with COVID-19 (P < 5×10–8), identified as single-nucleotide polymorphisms (SNPs), were chosen as candidate markers. These SNPs underwent further refinement through clumping with an r2 cutoff of 0.001 within a 10 Mb window to generate independent variables (IVs). The MR-Egger model’s intercept was utilized to estimate directional pleiotropy. Heterogeneity was assessed using Cochran’s Q test and I2 statistics (P < 0.05 and I2 > 0.25) (16).

### 2.3 Functional annotation

To gain a deeper understanding of the genetic networks associated with instrumental variables linking SARS-CoV-2 infection and HNC, we conducted a Functional Annotation analysis using the DAVID platform (https://david.ncifcrf.gov/). The genes corresponding to instrumental variables reflecting differential predisposition to SARS-CoV-2 infection and utilized in MR analysis have served as inputs for examining associated Gene Ontology (GO) terms and Encyclopedia of Genes and Genomes (KEGG) pathways in the categories of molecular functions, biological processes, and cellular components. For each gene, its participation in various biological pathways, including those related to metabolism, signaling, and diseases, was investigated. It is important to note that functional annotation serves primarily as a method to explore the role of each individual gene (e.g., determining which pathway or cellular process a gene is involved in) rather than assessing the function of the entire group of genes. Therefore, the annotation p-value or overlap percentage should be used for reference purposes only.

### 2.4 Knowledge-based analysis

We hypothesize that the genetic predisposition to milder forms of COVID-19 may reflect the state of the immune system, which serves as protection against HNC. To validate this hypothesis, we conducted an extensive data mining analysis using an AI-powered literature-data mining tool from AIC LLC (https://www.gousinfo.com/en/advancedsearch.html). We meticulously examined all extracted references and statements related to immune cells and biological processes concerning SARS-CoV-2 infection and HNC. This involved a thorough assessment of expression levels and functional states of each molecule of interest in NK cells, T cells, cytotoxic T cells, helper T cells, and their participation in innate and adaptive immune responses, checkpoints, and immunosuppression. We took precautions to ensure the extracted relationships were of high quality by eliminating any spurious or indirect associations.

## 3 Results

### 3.1 MR analysis results of SARS-CoV-2 infection on HNC

To investigate the causal impact of SARS-CoV-2 infection on HNC, we utilized three MR techniques: IVW (inverse variance weighted), WM (weighted median), and MR-Egger. In the susceptibility dataset for SARS-CoV-2 infection, a total of 14 instrumental variables (SNPs) were selected. The causal effects of SARS-CoV-2 infection on HNC, as determined by each MR technique, are summarized in **Table 1**.

**Table 1:** Causal effect of SARS-CoV-2 infection on head and neck cancers.

Exposure	Outcome	Method	#IV	b (se)	OR [95%CI]	p-value
SARS-CoV-2 infection	HNC	IVW	14	-0.651 (0.204)	0.52 [0.35–0.78]	1.42E-03
SARS-CoV-2 infection	HNC	WM	14	-0.515 (0.281)	0.60 [0.34–1.04]	0.066
SARS-CoV-2 infection	HNC	MR-Egger	14	-0.308 (0.393)	0.74 [0.34–1.59]	0.449

IVW, inverse variance weighted; WM, weighted median; OR, odds ratio; CI, confidence interval; #IV, number of instrumental variables.

Specifically, the IVW MR results suggest a significant association between SARS-CoV-2 infection and a reduced risk of HNC, with an odds ratio (OR) of 0.52. This implies that individuals with COVID-19 infection have an almost twice lower risk of developing HNC than the average population-wide risks of HNC, and this finding is statistically significant (p-value = 1.42E-03).

The other two MR methods, WM and MR-Egger, also yielded negative causal effects, indicating ORs of 0.60 and 0.74, respectively. However, these results have not reached statistical significance (p-values = 0.066 and 0.45, respectively).

**Figure 1:**
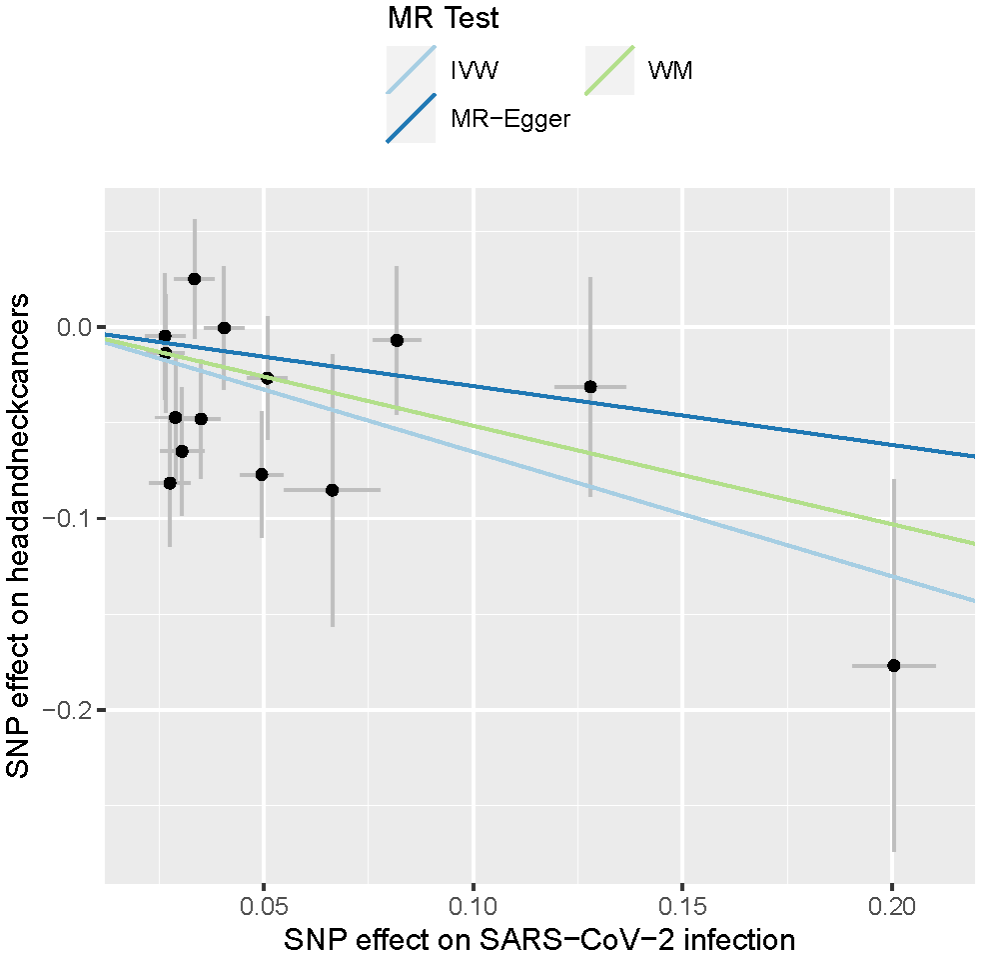
Causal effects of SARS-CoV-2 infections on head and neck cancers. The lines depict the effect sizes of the MR analysis. IVW, inverse variance weighted; WM, weighted median.

In summary, the results outlined in **Table 1** indicate that a less severe manifestation of SARS-CoV-2 infection may provide protection against the development of HNC. The significance of this protective effect varies depending on the statistical methods employed, with the IVW method yielding the most significant outcome. All three methods—IVW, WM, and MR-Egger—indicate a negative point estimate for the effect of SARS-CoV-2 infection on HNC, suggesting a consistent direction of association despite differences in significance. The heterogeneity analysis did not show any significant heterogeneity (P > 0.05), and there was no evidence of pleiotropy in this MR analysis (P > 0.05). The consistency across methods, along with non-significant results for heterogeneity and pleiotropy tests, strengthens the case for a potential causal effect, even if the WM and MR-Egger methods lack the power to confirm this definitively. These findings are also visually depicted in **Figure 1**.

### 3.2 MR analysis results of the effects of severe COVID-19 on HNC

**Figure 2:**
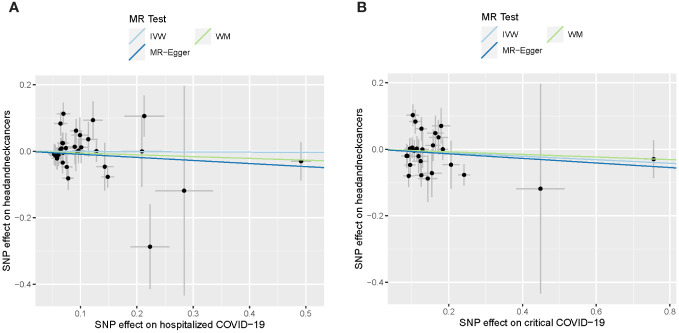
Causal effect of severe COVID-19 on head and neck cancers. **(A)** Causal effect of hospitalized COVID-19. **(B)** Causal effect of critical COVID-19. The lines depict the effect sizes of the MR analysis. IVW, inverse variance weighted; WM, weighted median.

For severe forms of COVID-19, including hospitalized and critical COVID-19, no apparent associations with the risk of developing HNC were detected (**Table 2**). Despite consistently showing a slight decrease in the risk of HNC (ORs ranging from 0.91 to 0.99), none of these findings reached statistical significance (p-value > 0.38). The results are also depicted graphically in **Figure 2**.

**Table 2:** Causal effect of Hospitalized COVID-19 on head and neck cancers.

Exposure	Outcome	Method	#IV	b (se)	OR [95%CI]	p-value
Hospitalized COVID-19	HNC	IVW	32	-0.007 (0.084)	0.99 [0.84–1.17]	0.932
Hospitalized COVID-19	HNC	WM	32	-0.054 (0.107)	0.95 [0.77–1.17]	0.614
Hospitalized COVID-19	HNC	MR-Egger	32	-0.092 (0.153)	0.91 [0.68–1.23]	0.549
Critical COVID-19	HNC	IVW	27	-0.053 (0.060)	0.95 [0.84–1.07]	0.382
Critical COVID-19	HNC	WM	27	-0.039 (0.068)	0.96 [0.84–1.10]	0.569
Critical COVID-19	HNC	MR-Egger	27	-0.069 (0.109)	0.93 [0.75–1.16]	0.534

IVW, inverse variance weighted; WM, weighted median; OR, odds ratio; CI, confidence interval; #IV, number of instrumental variables.

The heterogeneity analysis did not support the existence of heterogeneity (heterogeneity P > 0.05). There was no evidence supporting the pleiotropy in the MR analysis between SARS-CoV-2 infection and head and neck cancers (P > 0.05).

### 3.3 Functional annotation

The instrumental variables (IVs) used in the MR analysis for SARS-CoV-2 infection and HNC are located near 14 genes, including *OAS1, LOC107985887, BCL11A, DPP9, LOC107984685, LINC02326, MUC4, NXPE3, IFNAR2, LZTFL1, LOC105372437, NAPSA, LOC105376622, LOC107986082*, and *SLC6A20*. To deepen our understanding of the link between the mechanisms driving susceptibility to SARS-CoV-2 infection and HNC, we conducted a Functional Annotation analysis using the DAVID platform (https://david.ncifcrf.gov/). This analysis revealed that these genes are involved in a total of 14 Gene Ontology (GO) terms and/or KEGG pathways, the majority of which are associated with processes relevant to HNC, as outlined in **Table 3**. These findings support the reliability of the IVs used in the MR analysis conducted in the course of this work. Most of these pathways, in addition to their association with SARS-CoV-2 infection and HNC, also show a connection to the immune response. Therefore, we carried out an exhaustive literature-based exploration of data on the connections between SARS-CoV-2 infection, the immune system, and the development of HNC, as elucidated in the following section. It’s important to mention that certain genes among the 14 are not appear in the functional annotation results. Further investigation is required to establish their connection with HNC.

**Table 3:** Functional annotation analysis results of the 14 genes linking SARS-CoV-2 infection to head and neck cancers.

Term	Genes	% Overlap	P-Value
GO:0043129:surfactant homeostasis	NAPSA, OAS1	14.29	0.0065
GO:0035458:cellular response to interferon-beta	IFNAR2, OAS1	14.29	0.01
KW-0964:Secreted	NXPE3, IFNAR2, NAPSA, OAS1, MUC4	35.71	0.01
GO:0060337:type I interferon signaling pathway	IFNAR2, OAS1	14.29	0.02
hsa05162:Measles	IFNAR2, OAS1	14.29	0.032
GO:0098586:cellular response to virus	IFNAR2, OAS1	14.29	0.034
hsa05160:Hepatitis C	IFNAR2, OAS1	14.29	0.037
GO:0009615:response to virus	IFNAR2, OAS1	14.29	0.039
hsa05164:Influenza A	IFNAR2, OAS1	14.29	0.039
hsa04621:NOD-like receptor signaling pathway	IFNAR2, OAS1	14.29	0.043
hsa05169:Epstein-Barr virus infection	IFNAR2, OAS1	14.29	0.047

### 3.4 SARS-CoV-2 infection–immune system-HNC pathway

**Figure 3:**
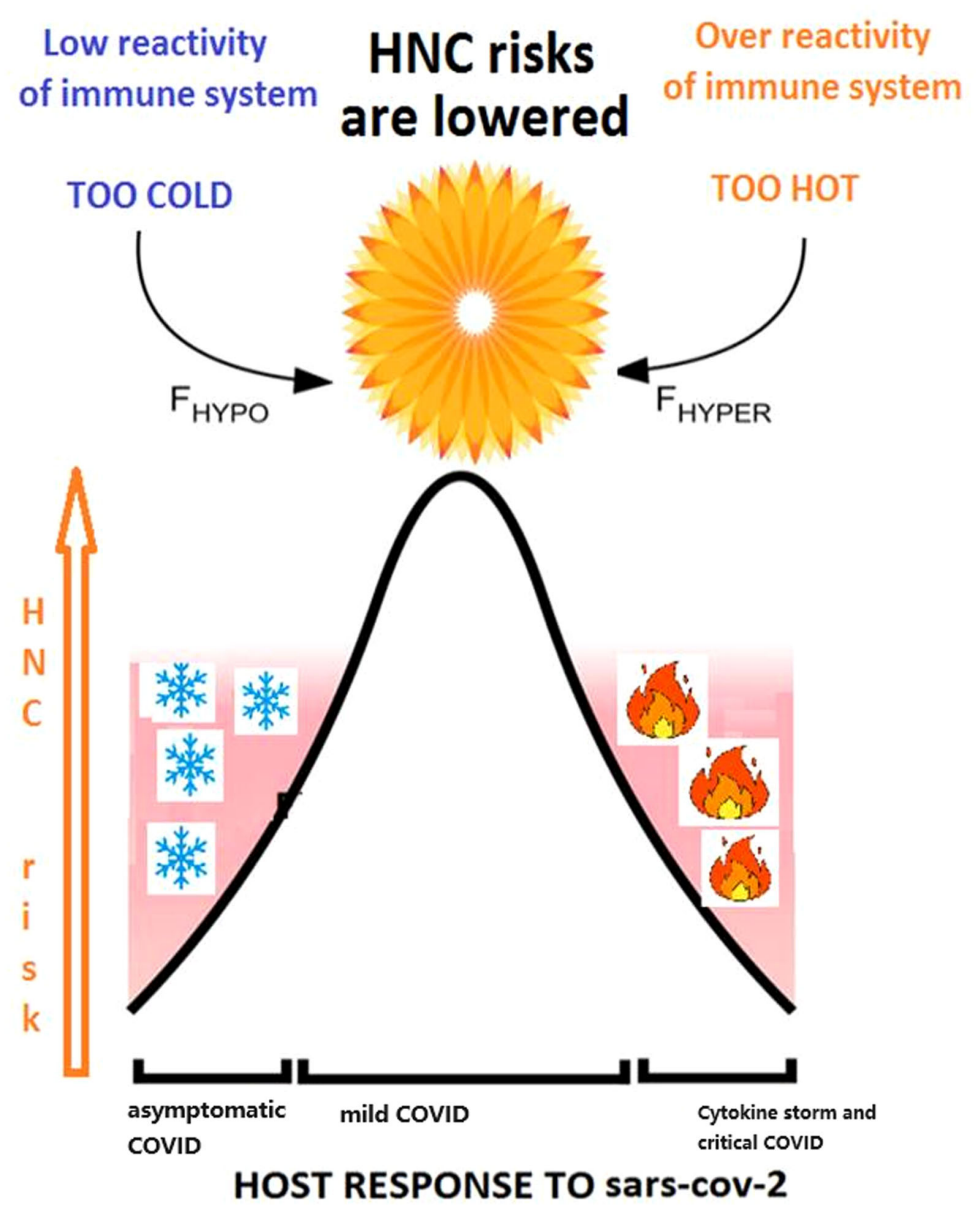
The relationship between response of immune system to COVID-19 and HNC cancer risk is reflected in Goldilocks principle.

By utilizing literature data mining powered by artificial intelligence, we deduce that a genetic predisposition to contracting milder form of COVID-19 may indicate an adequate steady-state of the immune system reactivity. This state is distinct both from the “cytokine storm” reactions driving patient deterioration in severe and critical cases of COVID-19 and from the lack of immune system reaction resulting in asymptomatic COVID-19, which typically remains undiagnosed. When a patient is genetically predisposed to maintain an optimal state of the immune system, resulting in the contraction of a milder form of COVID-19, the likelihood of developing HNC in this individual is lower compared to those with a genetic predisposition to either extreme state, such as anergy or cytokine storm. We depict our findings using a diagram as shown in **Figure 3**.

## 4 Discussion

While the impact of COVID-19 on the well-being of patients with HNC is undeniable, the majority of studies conducted thus far have focused on the indirect effects of the SARS-CoV-2 pandemic on the HNC population, particularly through delays in the diagnosis and treatment of this condition (7, 8). In contrast, our study has centered on investigating the relationship between genetic predisposition to various forms of COVID-19 and the risks of HNC. Surprisingly, we demonstrate that a predisposition to a milder, yet detectable, form of the disease may potentially act protectively, mitigating the risk of HNC development later in life.

When employing the IVW method, we observed a noteworthy association, signifying that contracting a symptomatic yet mild SARS-CoV-2 infection is significantly linked to a reduced risk of HNC (OR=0.52; p-value = 1.42E-03). The heterogeneity analysis did not indicate the presence of heterogeneity (P > 0.05). Additionally, there was no evidence supporting the potential for pleiotropy in this MR analysis (P > 0.05). Therefore, the assumptions for MR analysis using the IVW method are likely valid, indicating that the IVs are not violating the conditions necessary for causal inference (no pleiotropy and consistent effects across IVs). The significant result from the IVW method suggests a strong overall association when averaging the effects of the IVs. Although the WM and MR-Egger results are not statistically significant, all three methods show a consistent negative point estimate, and their confidence intervals overlap with those from the IVW method. This suggests that while the point estimates are similar, the precision varies. The IVW method generally has the highest statistical power among the three methods, leading to a significant result, while the WM and MR-Egger methods have less power, making it harder to achieve statistical significance with the same set of IVs and effect sizes (15). Notably, no discernible connection was found between genetic predisposition to severe forms of COVID-19 and the risk of HNC.

The importance of the steady-state alertness of the immune system for defense against viral infections is well-known (17). Either over- or under-activation of the innate immune response may be detrimental to infection clearance, with a carefully measured response required for the realization of a “Goldilocks” just-right scenario, or a “balancing act” (18). Recently, the importance of a well-balanced immune response has also been emphasized for the control and clearance of SARS-CoV-2 (18). Naturally, the same principle has found application in anti-tumor responses, for example, those driven by tumor-infiltrating lymphocytes (TILs) (19, 20).

The findings of our study, which were pleasantly surprising, neatly align with the “Goldilocks theory” of immune response. They indicate the involvement of 14 genetic loci associated with SARS-CoV-2 infection as contributors to the antitumoral maintenance of human tissues. These genetic loci correlate with 11 Gene Ontology (GO) terms/KEGG pathways predominantly associated with the antiviral immune response. Many of these genes have already been extensively described from a functional standpoint. For instance, genes *IFNAR2* and *OAS1*, profiled through their respective genetic variants rs2834158 and rs10774673, encode integral components of the type I interferon signaling pathway (21), which is crucial in the antiviral response. Additionally, they play a role in the NOD-like receptor signaling pathway, which significantly influences the development and progression of HNC and presents a potential therapeutic target in this context (22–24).

Another pathway highlighted by our analysis involves N-linked glycosylation, encompassing *NXPE3, IFNAR2, NAPSA, SLC6A20*, and *MUC4* genes through a set of genetic variations rs2290859, rs2834158, rs676314, rs73062389, and rs2260685, respectively. N-linked glycosylation plays a pivotal role in the context of HNC, impacting tumor aggressiveness and fostering cancer progression (25). Additionally, it has the potential to modulate chemotherapeutic (26) and immunotherapeutic (27) efficacy. These findings not only validate the use of instrumental variables (IVs) in the MR analysis but also offer insights into how the genetic predisposition to mild COVID-19 infection may influence the risk and prognosis of HNC.

When the immune system is impaired, one of its consequences is an increased susceptibility to severe infections, and another is the non-clearance of viral infections or their asymptomatic carriership. Individuals with compromised immune systems may also display a reduced response to vaccines, emphasizing the importance of additional protective measures (28). Immunosuppression heightens the likelihood and severity of HNC, with lactic acid-induced immunosuppression mediated by GPNMB and CD44 in the tumor microenvironment contributing to cancer progression. The effects of immunosuppression are nonspecific; they do not depend on its etiology. For example, prolonged immunosuppression in inflammatory bowel disease also increases the risk of developing HNC (29, 30). In the case of asymptomatic disease, which is expected to be common in adult COVID-19 controls according to recent epidemiological evaluations (31, 32), the state of immunosuppression may be present at the time of infection and is due to various genetic or environmental causes. Indeed, the epidemiology of the first two waves of COVID-19, which occurred before the advent of anti-SARS-CoV-2 vaccination, seems to reflect a mix of asymptomatic cases of SARS-CoV-2 with cases of non-exposure rather than true innate resistance to SARS-CoV-2 infection or pre-existing B- or T-cell immunity. On the other hand, when the SARS-CoV-2 disease is severe or critical, the function of natural killer (NK) cells is adversely affected, leading to reduced cytotoxicity and impaired antiviral responses, thereby compromising the immune system’s ability to combat tumor cells and potentially worsening the severity of the disease (33–35).

While the effects of the genetic predisposition to the differential course of SARS-CoV-2 disease on HNC and other tumors are challenging to prove in any kind of RCT, our findings definitely highlight the necessity for a longitudinal investigation into the differential risks of HNC and other cancers in patients with a documented course of COVID-19 or its carriership.

This study has several limitations that warrant further investigation in the future. Firstly, it is one of the few studies to observe an inverse causal relationship between mild COVID-19 and HNC at a genetic level. However, to validate this finding, clinical data collection and analysis are necessary. Secondly, the participants in the GWAS datasets were exclusively of European origin. Therefore, it is essential to examine data from other racial groups to corroborate the findings of this study.

## 5 Conclusion

The results of this study suggest that individuals with a genetic inclination for milder forms of COVID-19 may have a lower likelihood of developing HNC. However, the research does not establish a link between genetic predisposition to severe COVID-19 and the risk of HNC. Additionally, the study identifies 14 genes associated with milder COVID-19 that might play a role in mediating the protective impact against HNC.

## Data availability statement

Publicly available datasets were analyzed in this study. This data can be found here: https://www.covid19hg.org/results/r7/, https://storage.googleapis.com/finngen-public-data-r9/summary_stats/finngen_R9_J10_COPD.gz.

## Ethics statement

The publicly accessible summary results of genome-wide association studies (GWAS) utilized in this study were obtained from reputable sources, including the COVID-19 Host Genetics Initiative (HGI) and FinnGen R9. The original studies responsible for generating these datasets obtained appropriate ethics approvals and consent forms from the participants. The studies were conducted in accordance with the local legislation and institutional requirements. The participants provided their written informed consent to participate in this study.

## Author contributions

BH: Conceptualization, Writing – original draft, Writing – review & editing, Data curation, Formal analysis, Supervision, Visualization. MS: Formal analysis, Writing – original draft, Writing – review & editing, Methodology, Resources, Software. YZ: Methodology, Writing – original draft, Writing – review & editing, Data curation, Validation, Visualization. AB: Data curation, Visualization, Writing – original draft, Writing – review & editing, Conceptualization. HC: Conceptualization, Data curation, Writing – original draft, Writing – review & editing, Methodology, Resources. SL: Writing – original draft, Writing – review & editing, Software, Supervision. XS: Writing – original draft, Writing – review & editing, Formal analysis, Methodology. LH: Formal analysis, Writing – original draft, Writing – review & editing. JF: Writing – original draft, Writing – review & editing, Funding acquisition, Investigation, Project administration, Resources. ML: Funding acquisition, Investigation, Writing – original draft, Writing – review & editing, Conceptualization, Methodology.
